# Review of Books Received

**Published:** 1861-12

**Authors:** 


### REVIEW OF BOOKS RECEIVED

*Medical Jurisprudence. By* Alfred Swaine Taylor, M. D., F. R. S., *Fellow of the Royal College of Physicians; Hon. M. D. Univ. St. Andrews; Member of the Royal College of Surgeons; and Professor of Medical Jurisprudence and Chemistry in Guy's Hospital. Fifth American from the Seventh Revised London Edition. Edited, with additions, by* Edward Hartshorne, M. D., *one of the Surgeons to the Pennsylvania Hospital. Philadelphia:* Blanchard & Lea, 1861.

Since the first appearance of this work in 1844, there have issued from the press fifteen thousand seven hundred and fifty copies. This is proof of its practical utility to the members of the Medical and Legal Professions. This unparalleled encouragement has no doubt greatly stimulated the effort to maintain the work on a level with the progress of Medical and Legal Knowledge. In 1860, the Royal College of Physicians, and the Society of Arts, conferred on the author the quinquennial prize, under the will of the late Dr. Swiney, of one hundred pounds, and a silver vase of like value. There is perhaps no branch of medical knowledge so greatly neglected by active practitioners, as medical jurisprudence, and yet its important influence is often manifest in cases where testimony is given in open court, reflecting quite disparagingly upon the intelligence of the physician, even in strictly medical points. We must close our short notice of this book, which needs no recommendation from us, by advising our readers to not only place it upon the shelves of their libraries, but more especially to carefully peruse the many interesting chapters it contains, upon Insanity, Abortion, Pregnancy and Delivery, Rape, Wounds, Poisons, &c., &c. For sale in this city by Breed, Butler & Co.. 188 Main street.

*The Principles and Practice of Obstetrics. By* GUNNING S. BEDFORD, A. M., M. D., *Prof. of Obstetrics, the Diseases of Women and Children, and Clinical Obstetrics, in the University of New York; author of " Clinical Lectures on the Diseases of Women and Children," Illustrated by four Colored Lithographic Plates and ninety-nine Wood Engravings. 8vo. pp.* 763. *New York:* SAMUEL S. & WILLIAM WOOD, 389 *Broadway,* 1861.

Perhaps in no department of medicine has greater advances been made during the last few years, than in the field of Obstetrics; many new views in pathology have been fully established, and new modes of practice justified by results highly encouraging and satisfactory. We are happy to have presented a book upon the Principles and Practice of Obstetrics, which discusses some important topics, not in any way referred to in our former standard works on obstetrics; one also from which may be learned the most recent and correct views of pathology and treatment.

This book will be found a most complete, scientific, admirably arranged carefully considered work, upon obstetrics. In it the profession have presented them a practical treatise which fully developes the phenomena of parturition as it occurs in the lying-in room. The subject of labor, its divisions, its mechanism and management, its determining cause, together with the forces engaged in the expulsion of the child, the treatment of the puerperal woman, and her new-born infant. Flooding, both anti-partum and post-partum, placenta prævia, puerperal fever, puerperal mania, anæsthetics, have all been considered with great care, and the fullness their importance demands. The same may be said of the numerous questions connected with menstruation, reproduction, pregnancy, fœtal nutrition, puerperal convulsions, uremia, and many other kindred topics.

Manual, Instrumental and Premature Artificial Delivery, have received attention and been discussed fully and impartially. This book on obstetrics will rank as the highest authority in this department, and every physician who would conform his views and practice, to the teachings of correct observation, will act wisely in adding this book to his library.

For sale in Buffalo by BREED, BUTLER & Co., 188 Main street.

*Physician's Pocket Memoranda for* 1860. *By* C. H. CLEAVELAND, M. D., *Cincinnati, Ohio.*

This is truly a valuable pocket companion. It contains a list of medicines minutely classified. Instructions in writing prescriptions, abbreviations, &c., directions in cases of accidents and emergencies. The division on poisons and antidotes is very valuable. The book contains instructions for post-mortem examinations, preservation and embalming. The body of the book is conveniently arranged for physician's memoranda. This is one of the most convenient, compact and useful diaries yet published. Every physician should provide himself with one.

Price $1.00. Address Dr. C. H. CLEAVELAND, Cincinnati, Ohio.

*Dentist's Memorandum—A Book of engagements and manual of ready reference, much enlarged and improved. By* C. H. CLEAVELAND, M. D., *Cincinnati, Ohio.*

This work is so arranged that every week-day throughout the year can be used for keeping the accounts of all operations performed. Each hour in the day, from eight in the morning until five in the afternoon, is divided into tabular form for recording engagements. Dollar and cent lines are ruled so as to charge for operations against each engagement, with the name of the person for whom the operation is performed, together with a large number of valuable receipes for Mouth Washes, Tooth Powders, Tooth Pastes, Washes for Inflamed Gums, Zinc Paste for filling Teeth, ("Os-Artificial," "Os-teoplastic," etc.,) Toothache Drops, Gold and Silver Solders, Varnishes for Models, treatment for Neuralgia, Exposed Nerves and Sensitive Dentine, Hemorrhage, Tempering Instruments, Fungus Growths, Filling Cavities, etc., etc. Making altogether, in a small and convenient form, one of the most convenient, practical, and useful works, so far at least as we are able to judge of the wants of the dental profession.

Price $1.00. Address C. H. CLEAVELAND, Cincinnati, Ohio.

---

